# Supplementary material for: Population-based identification and temporal trend of children with primary nephrotic syndrome: The Kaiser Permanente nephrotic syndrome study
Source: PLoS One. 2021 Oct 14;16(10):e0257674. doi: 10.1371/journal.pone.0257674 (PMC8516311; doi:10.1371/journal.pone.0257674)
Supplement: S1 File — (DOCX) [file pone.0257674.s001.docx]

# Supplementary Document. Approach to physician adjudication for primary nephrotic syndrome and presumed etiology.

Pediatric patients confirmed with nephrotic syndrome demonstrated evidence of any of the following in their electronic medical records.

1. Lab result indicating nephrotic range proteinuria
   1. Urine Albumin-to Creatinine Ratio (ACR) >3500 ug/mg OR
   2. Urine Protein-to-Creatinine Ratio (PCR) >2 mg/mg OR
   3. 24-hour Urine Protein excretion >40/mg/m^2^/hr, after adjusting for the child’s height and weight
   4. Urine protein dipstick: >2+
2. Consistent duration of proteinuria
3. Presence of kidney biopsy

The following exclusion criteria were applied if found during physician adjudication of medical records:

1. Diagnosed nephrotic syndrome or evidence of nephrotic range proteinuria that was attributed to multiple myeloma, cancer, hepatitis C, systemic lupus, preeclampsia, diabetes mellitus, Wegener’s syndrome, or other secondary etiology
2. Diagnosed nephrotic syndrome or nephrotic range proteinuria developing after kidney or other organ transplantation
3. Biopsy report of diabetic nephropathy in patients with diagnosed focal segmental glomerulosclerosis (FSGS)
4. Documented nephrotic range proteinuria but no confirmation of nephrotic syndrome in manual review of medical records
5. Post-nephrectomy nephrotic range proteinuria without evidence of a biopsy
6. Nephrotic range proteinuria during pregnancy that does not persist after pregnancy
7. Transient nephrotic proteinuria with no biopsy, nephrology consultation, or clinical follow-up data

Approach for assigning presumed cause of adjudicated nephrotic syndrome:

1. When available, use biopsy diagnosis.
2. When multiple presumed causes are found in medical records, use the treating nephrologist-assigned diagnosis.
3. When no biopsy result is available, use other clinical data, if available (e.g. nephrologist notes, transfer notes from other facility).
4. All causes determined are "presumed," unless definitive biopsy or blood test result is available.

**Supplementary Fig. S1.** Mean age- and sex-adjusted 1-year incidence of primary nephrotic syndrome among all pediatric Kaiser Permanente Northern California members without diabetes mellitus between 1996 and 2012, directly standardized to the 2010-2012 population.

**Supplementary Fig. S2.** Mean 1-year incidence* of primary nephrotic syndrome among all pediatric Kaiser Permanente Northern California members without diabetes mellitus between 1996 and 2012, stratified by age group.

*Note: Lines between point estimates in figure are displayed for clarity and do not reflect calculated rates.

**Supplementary Fig. S3.** Mean 1-year incidence* of primary nephrotic syndrome among all pediatric Kaiser Permanente Northern California members without diabetes mellitus between 1996 and 2012, stratified by sex.

*Note: Lines between point estimates in figure are displayed for clarity and do not reflect calculated rates.
