# Supplementary material for: Population-based identification and temporal trend of children with primary nephrotic syndrome: The Kaiser Permanente nephrotic syndrome study
Source: PLoS One. 2021 Oct 14;16(10):e0257674. doi: 10.1371/journal.pone.0257674 (PMC8516311; doi:10.1371/journal.pone.0257674)
Supplement: Attachment — Submitted filename: Review PLOS ONE.docx [file pone.0257674.s003.docx]

Review Population based… for Plos One

1. Was funded by Brin Wojcicki, so why say ‘Grant Not Applicable’. Please give url of Funder
2. Line 308: What is the percentage of insured versus uninsured population?
