# Supplementary material for: Population-based identification and temporal trend of children with primary nephrotic syndrome: The Kaiser Permanente nephrotic syndrome study
Source: PLoS One. 2021 Oct 14;16(10):e0257674. doi: 10.1371/journal.pone.0257674 (PMC8516311; doi:10.1371/journal.pone.0257674)
Supplement: Attachment — Submitted filename: Parikh_Pediatric_NS_PLOS_Response_09Apr2021.docx [file pone.0257674.s004.docx]

| Reviewer 1 Comments | Response |
| --- | --- |
| The authors estimate and characterize the population incidence of pediatric primary NS in a large, integrated healthcare delivery system through structured review of available data within electronic health records (EHR) and associated health system data sources. They conclude that this population-based identification of children with primary NS leveraging electronic health records can provide a unique approach and platform for describing the natural history of NS and identifying determinants of outcomes in children with primary NS. The study is interesting, however, there are some concerns. | We thank the Reviewer for their comments and have done our best to address the issues raised. |
| 1) The supporting literature for the definition of NS in this study should be clearly stated in the text. | We thank the Reviewer for this suggestion, and we have added a clarification to the revised Methods section along with a textbook reference. The specific definition used in this study based on proteinuria cutoffs has not been previously used and was designed for the purposes of this study to be highly specific for potential nephrotic syndrome, especially when using electronic health records data alone. Of note, the proteinuria thresholds described in the added Supplemental Document are more in line with the clinical definition, and these criteria were used by reviewers when manually adjudicating cases in the context with other clinical notes and related data in available medical records. |
| 2) The indications for renal biopsy in NS in the population in this study should be presented. The indications for renal biopsy may have a significant impact on the data presented in this study. | We discuss the issue of potential indications for a kidney biopsy in the revised limitations section. In clinical practice, biopsies are only performed in children when NS is persistent, not responsive to treatment, and considered idiopathic. In our study, we did not require biopsies for children to be eligible for the cohort; instead we focus on diagnosis codes, documented proteinuria, and nephrology notes to determine NS with a presumed etiology. Biopsy reports, if available, were used to confirm the presumed etiologies but were not necessary for inclusion in our cohort of NS patients. Although this approach may result in some misclassification of the presumed etiology, we believe this is likely minimal as more than 80% of patients classified as having presumed FSGS and MN had a kidney biopsy result. In addition, we believe that the validity of overall NS cases is not affected by the availability of biopsy results, as NS in children in typical clinical practices is empirically diagnosed based on documented proteinuria and associated symptoms/signs, along with demographic information. |
| 3) Many pediatric NS patients have MCD, and a significant proportion of these patients do not have renal biopsy. According to the method used by the authors in this study, it should be possible to identify patients with NS who have not undergone renal biopsy. In pediatric patients, the population without renal biopsy is also important and should be included in the analysis. | We appreciate the Reviewer’s point and note that we have included the proportion of patients with MCD in the analysis as the presence of a kidney biopsy was not a required inclusion criterion.  To further illustrate the Reviewer’s point, we note many more biopsies were performed at KPNC in patients with FSGS and MN than in patients with MCD. |
| 4) In pediatric NS, the term "idiopathic" is often used. The authors use the term "primary" instead of "idiopathic". This is correct if the disease is confirmed as "primary" by renal biopsy. On the other hand, the rate of renal biopsy among 179 primary NS is shown to be 40.2%. This statement is strange. How can they distinguish between MCD, FSGS, and MN when no renal biopsy was performed? Does this mean that re-biopsy data was collected in some cases? The authors should clearly explain this point and improve the manuscript to avoid misleading the readers. There is a significant problem with this database if they are diagnosing FSGS and MN without performing renal biopsy. | We thank the Reviewer for these comments and wish to clarify our assignment of etiology. We refer to all etiologies as “presumed” in the absence of a kidney biopsy. For some pediatric patients who transferred from other non-KPNC facilities, biopsies were not re-done at KPNC, and the diagnosis of FSGS or MN was carried over from their previous health records and/or mentioned in the treating nephrologist notes. Unfortunately, we had incomplete ascertainment of biopsy reports from other facilities. Additionally, invasive biopsies are not often performed in children with minimal change disease or steroid sensitive nephrotic syndrome.  We have added this clarification to both the revised Methods text and the limitations section of the discussion. We have also added a Supplemental document with the chart review methodology and rules for assigning presumed etiology. |
| 5) There were 67 cases (37.4%) with missing data for renal function. A total of 67 cases (37.4%) with missing data for renal function is a serious limitation of this study. Why is this the case? Do many attending physicians not measure renal function in NS patients? The same is fact for serum albumin. The authors mention that we additionally performed targeted manual chart review by board-certified nephrologists for evidence of a clinical presentation consistent with NS which enhanced our specificity and reduced misclassification compared with reliance only on administrative diagnosis codes. Also, the authors described that all primary NS cases were manually confirmed through targeted manual medical records review by nephrologists, which is considered the gold standard for retrospective case identification and also demonstrated the suboptimal performance of diagnosis codes alone to identify children with NS. If so, I would guess that kidney function and serum albumin could be easily checked. The high incidence of missing data for renal function and serum albumin in NS studies is fatal. Appropriate improvements in the manuscript are desirable. If this is not possible, then the data extraction itself may be inappropriate, and the credibility of the data presented may be affected. | We appreciate the Reviewer’s comment, and we have updated the retrospective data search which include additional data that was incorporated into our database since the analysis was originally performed. In addition, we included laboratory results performed within 30 days after the confirmed NS date, to account for testing prompted by new proteinuria, a new NS diagnosis, or biopsy. This substantially reduced the missingness of kidney function and albumin to 24% and 32%, respectively. We then manually examined a sample of these cases to confirm that our database was not missing known tests and found that in these cases, testing was, in fact, not performed within our ascertainment windows, confirming no systematic database issues. In typical pediatric clinical practices, blood tests for serum creatinine and albumin may not be performed if the clinical presentation and documented proteinuria strongly supports an NS diagnosis that is subsequently managed effectively by treatment. Therefore, we respectfully disagree with the Reviewer and believe our data provide a more generalizable perspective on NS in a large community-based population. However, we have added this as a limitation and provide a potential explanation for the lack of available baseline data in this subset of children. |
| 6) The numbering of the figures is wrong. They do not match the text. | We thank the Reviewer for noticing and have corrected the referenced figure numbers. |
| 7) In Table 2, the number of kidney biopsies performed is not correct. MCD (30), FSGS (36), and MN (6) add up to only 72, while the total is 74. | We thank the Reviewer for noting this discrepancy and have edited Table 2 to reflect updated data. |
| Reviewer 2 Comments | Response |
| The authors describe Population-based identification and temporal trend of children with primary nephrotic syndrome:  1. The study was funded by Brin Wojcicki, so why say ‘Grant Not Applicable’. Please give url of Funder | We thank the Reviewer for this comment. Unfortunately, the Brin Wojcicki Foundation does not operate a website. |
| 2. Line 308: What is the percentage of insured versus uninsured population? | We thank the Reviewer for this question and clarify that our analysis only included insured individuals (i.e. members of the Kaiser Permanente Health Plan). Nearly all individuals receiving outpatient care at KPNC are insured members--uninsured individuals or non-members admitted to KPNC emergency departments were not included. |
