# Supplementary material for: Population-based identification and temporal trend of children with primary nephrotic syndrome: The Kaiser Permanente nephrotic syndrome study
Source: PLoS One. 2021 Oct 14;16(10):e0257674. doi: 10.1371/journal.pone.0257674 (PMC8516311; doi:10.1371/journal.pone.0257674)
Supplement: Attachment — Submitted filename: Parikh_Pediatric_NS_PLOS_Response_23Jun2021.docx [file pone.0257674.s005.docx]

| Reviewer 3 Comments | Response |
| --- | --- |
| This is an interesting and well thought out manuscript that uses the electronic health record to assess the incidence of nephrotic syndrome in pediatric patients in a well characterized and excellent health care system. | We thank the Reviewer for these kind comments. |
| 1. The number of patients is unclear. Table 1 is a bit confusing and it is unclear whether the number of patients is 365 or 179. I think a CONSORT diagram documenting the steps to reach the analytic sample would be helpful | We apologize for any confusion and kindly direct the Reviewer to Figure 1 which is a CONSORT diagram of the study cohort assembly. We have modified Figure 1 to show the additional exclusion of nephrotic syndrome from secondary causes to reach the final sample of 179 children with primary nephrotic syndrome. |
| 2. The definition of nephrotic syndrome is more appropriate for adults and not pediatric patients where the proteinuria threshold is >2 mg/mg creatinine | We appreciate the Reviewer’s comment and wish to clarify that children with potential nephrotic syndrome were initially identified using two criteria using data from our electronic health record and clinical laboratory database: 1) presence of a diagnosis code for nephrotic syndrome, or 2) evidence of nephrotic-range proteinuria (>3.5 mg/mg). These proteinuria thresholds were designed to be more specific for nephrotic syndrome especially among those without a corresponding diagnosis code for NS.  Patients meeting either of these criteria were then manually reviewed by a qualified physician using slightly lower clinical proteinuria thresholds (i.e., >2 mg/mg) along with other aspects of presentation, described in more detail in the Supplemental Document. Therefore, patients with proteinuria between 2-3.5 mg/mg were included if they also had a clinical syndrome consistent with nephrotic syndrome.  We acknowledge that this approach may contribute to underestimation of the apparent incidence of primary nephrotic syndrome reported in the study in favor of higher specificity for identified cases and have added this to the Discussion. |
| 3. The impact of race is not clearly presented. | We thank the Reviewer for this suggestion. In Table 2, we present the racial distribution overall and across presumed etiologies of primary nephrotic syndrome. Unfortunately, self-reported race is not well-captured in children and ~30% of the analyzed cohort has an unknown race. In this cohort, we can observe that children with FSGS are more likely to be Black or multiracial as has been previously described by others, and that most children of Asian or Pacific Islander descent with primary nephrotic syndrome have presumed minimal change disease. Aside from these observations, which we have now added to the Results section, we do not believe any additional analyses of incidence by race are possible given the small sample size per group. We have added this as a limitation to the Discussion. |
| 4. The authors might comment on the minor change in the incidence of nephrotic syndrome over time | We thank the Reviewer for this suggestion and have added another comment to the Discussion. From supplemental analyses, we observe that this increase may be driven by increases in incidence among children aged 6-11 years, and particularly among boys compared with girls. However, it is unclear whether this increase reflects a true increase in NS incidence in the underlying populations, or a change in practice patterns or population demographics over time. |
| 5. The authors might want to compare their findings to those in adults published in AJKD that was based on the same health care system. | We appreciate the Reviewer’s suggestion. As part of this same effort, we also identified and analyzed adult KPNC members with primary nephrotic syndrome using the same methodology. This analysis was recently published in JASN: (<https://jasn.asnjournals.org/content/early/2021/06/18/ASN.2020111583>).  We refer to the previously published AJKD study from Kaiser Permanente Southern California in the adult manuscript.  Although we agree that the efforts are comparable, we have focused the Discussion here on other studies among children and the implications for pediatric epidemiology. |
| Reviewer 4 Comments | Response |
| 1. The study presents the results of original research.  Nephrotic syndrome (NS) is a clinical diagnosis with proteinuria, hypoalbuminemia and odema. Although primary NS is relatively uncommon, it accounts for mortality and considerable morbidity in young children. The prognosis of NS in children correlates with the spectrum of responsiveness to steroid therapy, from steroid-sensitive NS (SSNS) to steroid-resistant NS (SRNS). SRNS is the most common acquired cause of end-stage renal disease (ESRD) in children. Kaiser Permanente (KP) in the Northern California region, provides an integrated healthcare delivery system caring for >750,000 children. Primary NS in the region is relatively uncommon, and no prior population-based research in the region has been carried out to study the incidence of primary NS in children. The incidence being unknown, both based on the clinical patterns and ethnic distribution despite the data being available in the EMRs and laboratory databases of the KP systems. The aim of this study was to estimate the incidence of primary NS in the region based on a retrospective laboratory and EMR data with a multicenter collaboration within the KP systems. Such data would help in improving population-level quality of care and thereby clinical outcomes. Additionally, would contribute to the development and implementation of system-wide efforts to improve complex delivery care systems for the illness. This research data despite its limitations of being retrospective and only 40% of the children having a renal biopsy, will facilitate infrastructure and processes that support the ability to accelerate large-scale change internally, as well as externally through successful multi-institutional collaborations within the Kaiser Permanente as seen in the paper. In conclusion the study does present results of original research work done in the region of northern California cared for by the Kaiser Permanente integrated healthcare delivery system and agree with the authors that the study can provide a unique approach and platform for describing the natural history of NS and identifying determinants of outcomes in children with primary NS. | We thank the Reviewer for summarizing the study and for their kind comments. |
| 2. Results reported have not been published elsewhere.  The results reported have not been published elsewhere as far as my search for the same has revealed. This data although retrospective does provide a comphrensive input into the incidence of pediatric nephrotic syndrome in the region split by clinical presentation and ethnic breakup. | We thank the Reviewer for their comments. |
| 3. Experiments, statistics, and other analyses are performed to a high technical standard and are described in sufficient detail.  The analysis is preformed to the needed technical standard and done in sufficent detail for the data that is available. All data are made fully available without restriction and the authors have made available a file of the minimal de-identified data set necessary to replicate the analyses in this manuscript. | We thank the Reviewer for their comments. |
| 4. Conclusions are presented in an appropriate fashion and are supported by the data.  The data is retrospective and collected from the EMRs as well as laboratory databases, from across the KP organization. There could be changes in the etiology and the racial presentation presented, since only 40% have biopsy data and there is no long-term follow-up reported after the initial diagnosis. Especially, that in 60% of the patients the diagnosis was entirely clinical and not supported with renal histology. Although ethnic differences in clinical presentations are shown, it would have been useful to have follow-up data on outcomes among these children with nephrotic syndrome which are unknown. Despite the higher incidence of NS among ethnics, outcomes may be significantly less complicated when compared between various clinical presentations. Within the above limitations the presentation is appropriately supported. | We thank the Reviewer for their comments. |
| 5. The article is presented in an intelligible fashion and is written in standard English.  Yes, the article is presented in an intelligible fashion and written in standard English with no flaws observed within the article. | We thank the Reviewer for their comments. |
| 6. The research meets all applicable standards for the ethics of experimentation and research integrity.  The research does meet with all the applicable standards for the ethics of experimentation and research integrity. The study was approved by the KPNC institutional review board, and waiver of informed consent was obtained due to the nature of this retrospective data-only study, with the use of de-identified data sets as reported. The authors have also made the relevant disclosures which do not conflict with the results of the study: ASG declares funding from the Brin Wojcicki Foundation. GMC declares grants from NIDDK, and Amgen, and fees from Ardelyx, AstraZeneca, Baxter, Cricket, DiaMedica, Gilead, Reata, Sanifit, Vertex, Satellite Healthcare, Angion, Bayer, ReCor and other disclosures from CloudCath, Durect, and Outset. All other authors have nothing to disclose. | We thank the Reviewer for their comments. |
| 7. The article adheres to appropriate reporting guidelines and community standards for data availability.  The manuscript does provide a clear and complete account of the research done. The reporting does clearly allow editors, peer reviewers and readers to understand what was done and how. The data can be clearly understood by a reader, replicated by a researcher, and used to make a clinical decision and included in a systematic reveiw with its limitations. | We thank the Reviewer for their comments. |
